# The Procyanidin C1-Dependent Inhibition of the Hydrolysis of Potato Starch and Corn Starch Induced by Pancreatin

**DOI:** 10.3390/molecules26206121

**Published:** 2021-10-11

**Authors:** Umeo Takahama, Sachiko Hirota

**Affiliations:** 1 Department of Dentistry, Kyushu Dental University, Kitakyushu 803-8580, Japan; 2 Sanyo-Gakuen College, Okayama 703-8501, Japan; sachiko_hirota@sguc.ac.jp

**Keywords:** amylose/procyanidin complexes, hydrolysis of liberated amylose, molecular weight of amylose, procyanidin C1, slower amylose hydrolysis

## Abstract

Procyanidins are contained in various foods, and their effects on starch hydrolysis have been reported. In Japan, black soybeans, which contain a trimeric procyanidin, procyanidin C1 (proC1), are cooked with rice and used to prepare dumplings. In this study, the effects of proC1 on the pancreatin-induced formation of reducing sugars and starch hydrolysis were studied using potato starch and corn starch. ProC1 inhibited both reactions; the inhibition was greater in potato starch than corn starch when added to heated potato starch and corn starch. When heated with proC1, its inhibitory effects decreased, especially in potato starch, suggesting the important role of proC1 itself for the inhibition of potato starch hydrolysis. ProC1 also inhibited the hydrolysis when added to heated, longer amylose (average molecular weight: 31,200), and the inhibition decreased when heated with the amylose. On the other hand, proC1 could not inhibit the hydrolysis when added to heated, shorter amylose (average molecular weight: 4500), but could when heated with the amylose, suggesting the important role of the degradation products of proC1 for the inhibition. We discuss the mechanism of the proC1-dependent inhibition of amylose hydrolysis, taking the molecular weight into account.

## 1. Introduction

Proanthocyanidins are oligomeric and polymeric flavan-3-ols. The flavan-3-ol units linked through the C4→C8 bond or the C4→C6 bond are called B-type proanthocyanidins. An ether bond between C2 and C7 is added to the B-type, and the proanthocyanidins are called A-type. The proanthocyanidins can be classified into three types depending on the hydroxylation patterns of the B-ring of the flavan-3-ol units, namely, propelargonidins, procyanidins, and prodelphinidins. Of the proanthocyanidins, procyanidins are commonly found in various foods [1,2].

Proanthocyanidins are of great interest in nutrition and medicine because of their antioxidant capacity, which has the potential to protect the human body from various diseases [3]. Adzuki beans (*Vigna angularis*), apples, black soybeans (*Glycine max* L.), sorghum, and so on are known as proanthocyanidin-rich foods. The benefits of the foods for human health have been recently reviewed [4,5]. If proanthocyanidins are effective at slowing down starch hydrolysis in the intestine, one of the causes of lifestyle diseases such as diabetes and adiposis might be alleviated by taking a starch-based food with proanthocyanidin-rich foods.

It has been reported that oligomeric and polymeric proanthocyanidins can bind to amylose, decreasing its digestibility [6,7,8,9]. In addition, a dimeric procyanidin, procyanidin B2, has been reported to be effective at slowing down amylose hydrolysis in nonglutinous rice and high-amylose rice cooked with adzuki beans [10,11,12], and procyanidin B2 has also been reported to be efficient at slowing down amylose hydrolysis when heated with potato starch and reagent amylose [10]. In these reports, however, the mechanisms of the slowdown have not been made clear. In relation to the above studies using adzuki beans, the inhibition of amylose hydrolysis by cyanidin 3-*O*-glucoside [13] and the cooperation of the cyanidin glucoside and procyanidins for the inhibition of amylose hydrolysis [14] have been reported using black soybeans. In these reports, it has been suggested that the inhibition by cyanidin 3-*O*-glucoside and procyanidins is due to their binding to amylose. 

In black soybeans, there is a trimeric procyanidin, procyanidin C1 (proC1), in addition to procyanidin B2 [15]. According to [6,7,8,9], it is expected that proC1 might more effectively interact with amylose than procyanidin B2 because of the difference in the three-dimensional structures of the two compounds. At present, however, the effects of proC1 on starch hydrolysis have not been reported. This paper deals with the proC1-dependent inhibition of the amylose hydrolysis of potato starch, corn starch, and amylose reagents with different molecular weights. The obtained results suggest that the inhibition of amylose hydrolysis by proC1 might be dependent on the molar ratio of procyanidin C1 and amylose. It is discussed that the combination of two amylose molecules by a proC1 molecule might contribute to the inhibition of amylose hydrolysis by proC1.

## 2. Results 

### 2.1. Effects of ProC1 on Reducing Sugar Formation

**Scheme 1:**
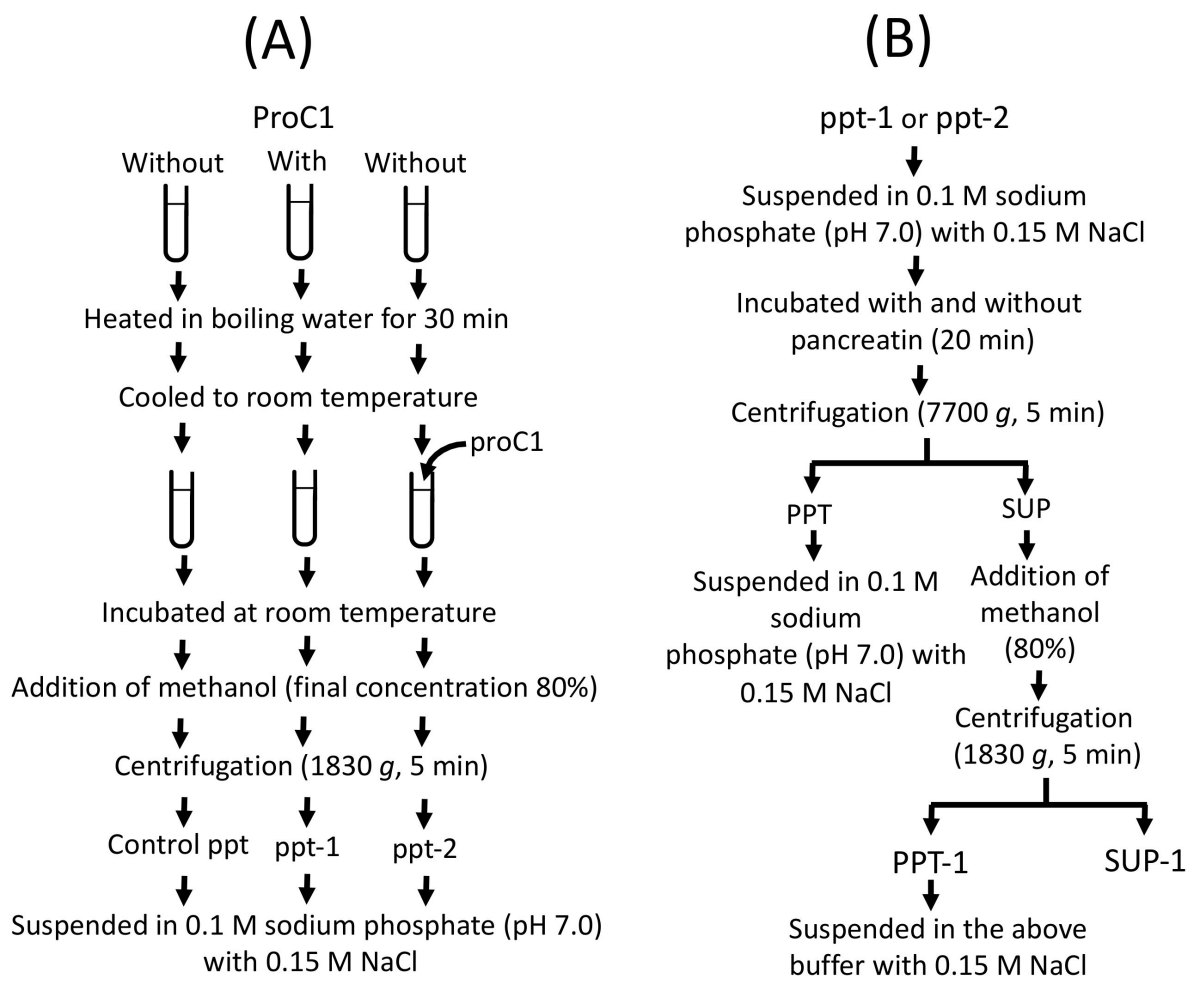
Preparations of samples used in this study. (**A**) Preparation of precipitates (ppts) to measure pancreatin-induced starch digestion. Each test tube contains potato starch, corn starch, Mw4500, or Mw31200 (10 mg/mL). The ppts were used to obtain the data for Figures 1 and 2, and for Tables 1, 2, and 4. (**B**) Preparation of precipitates (PPT and PPT-1) and supernatants (SUP-1) from ppt-1 and ppt-2 to estimate the amounts of proC1 in the fractions. The fractions were used to obtain data for Table 3 (For details, see Section 4.2).

Control ppt, ppt-1, and ppt-2 prepared from potato starch and proC1 (Scheme 1A) were suspended in a buffer solution with 0.15 M of NaCl to study the pancreatin-induced formation of reducing sugars. Their formation was nearly linear as a function of incubation time, as reported previously [10], and the formation was 8 ± 4 and 15 ± 5% (*n* = 3) slower in the ppt-1 and the ppt-2, respectively, than the control ppt. When compared in each pair of experiments, the reducing sugar formation was 7 ± 3% (*n* = 3) slower in the ppt-2 than in the ppt-1. These results indicate (i) that proC1 could inhibit the reducing sugar formation in potato starch, and (ii) that the effect was decreased by heating. The reducing sugar formation was 6 ± 4 and 12 ± 9% (*n* = 3) slower in ppt-1 and ppt-2, respectively, than in the control ppt prepared from corn starch. The formation in the ppt-2, however, was not significantly slower than that of the ppt-1 (6 ± 12%, *n* = 3) when compared in each pair of experiments. 

### 2.2. Effects of ProC1 on Starch Hydrolysis

**Figure 1:**
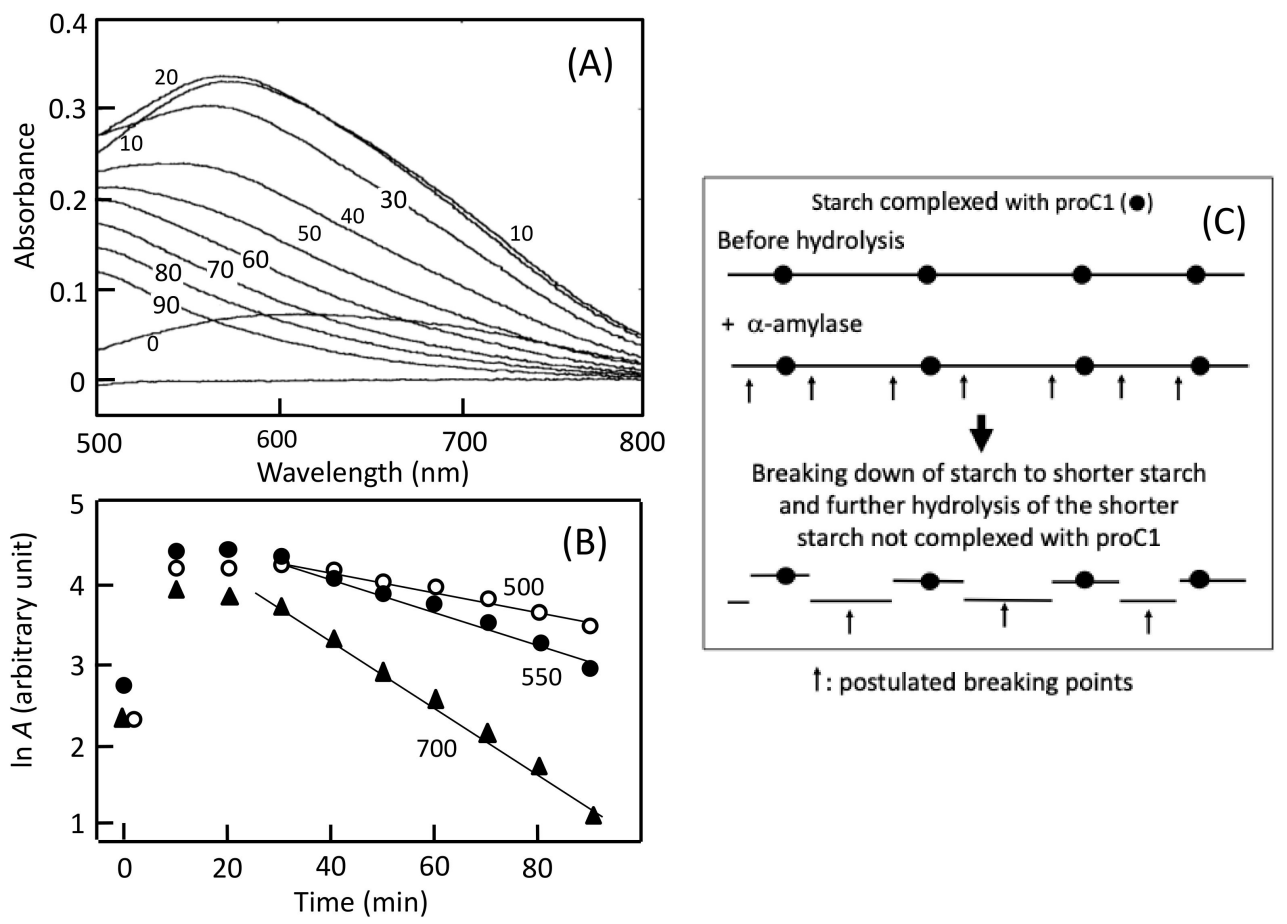
Pancreatin-induced hydrolysis of starch of control ppt of potato starch. The control ppt was prepared as described in Section 4.2 (Scheme 1A). The concentration of starch liberated and hydrolyzed by pancreatin was estimated using iodine. (**A**) Changes in absorption spectra of starch/iodine complexes as a function of incubation time. Numbers on traces: time (min) after the addition of pancreatin. (**B**) Semilogarithmic plots of absorbance changes. (○) 500 nm; (●) 550 nm; (▲) 700 nm. (**C**) Postulated breaking down of an amylose molecule complexed with proC1. A shorter starch combined with proC1 might be less hydrolyzable.

When the control ppt of potato starch was suspended in the buffer solution with 0.15 M of NaCl in the absence of pancreatin, water-soluble starch was found in the suspension, and the starch/iodine complexes had a broad peak around 600 nm (Figure 1A, trace 0). This indicates that water-soluble amylose might have diffused out from the ppt. The diffusing of amylose is possible because it is leached out from potato starch during heating [16], and leached amylose covers the surface during retrogradation [17,18]. It has been reported (i) that starch leached out from nonglutinous rice and glutinous rice during cooking covers the surface of rice grains during retrogradation [19,20], and (ii) that amylose and amylopectin on the surface are diffused out from the cooked nonglutinous rice and the glutinous rice, respectively, by suspending in a buffer solution [10].

The addition of pancreatin to the suspension of the control ppt resulted in the liberation of water-soluble starch from the ppt, and the liberated starch had a peak around 570 nm with a considerable absorbance around 700 nm when complexed with iodine (Figure 1A, trace 10). The absorption spectrum of the liberated starch was similar to that of the reagent amylose Mw4500 (28 glucose residues in average), which was dissolved in 0.1 M of sodium phosphate with 0.15 M of NaCl by heating in gently boiling water for 30 min, suggesting that the starch liberated from the control ppt mainly consisted of amylose as long as the starch was detected using iodine. It has been reported that a significant number of the amylopectin branches of potato and maize consist of 30 or fewer glucose residues [21,22]. The value suggests that when pancreatin induces the liberation of water-soluble amylopectin from the control ppt of potato starch, the chain lengths might be shorter than 30 glucose residues. This is deduced from models where the branches of amylopectin molecules in retrograded starch granules face the surface of the granules [17,18]. In fact, amylopectin liberated from cooked glutinous rice did not have any peaks in the wavelength range from 500 to 700 nm [10]. 

The amylose liberated by pancreatin during the 10 min of incubation was hydrolyzed (Figure 1A). The hydrolysis was observed as an absorbance decrease as a function of incubation time in the wavelength range from 500 nm to 800 nm. Accompanying the decrease, the absorption peak shifted to shorter wavelengths, suggesting that the liberated amylose molecules were hydrolyzed successively, decreasing their glucose chain lengths. The semilogarithmic plots of the absorbance decrease at 500, 550, and 700 nm suggest that the absorbance decreases were first-order-like reactions (Figure 1B). Pancreatin also induced the liberation of starch from ppt-1 and ppt-2 prepared using potato starch, and the changes in absorption spectra and decreases in absorbance at the above three wavelengths as a function of incubation time were essentially the same as those in Figure 1A,B, respectively, except for the rate. Furthermore, control ppt, ppt-1, and ppt-2 of corn starch were also hydrolyzed as in Figure 1A,B.

Table 1 shows that the half-life of the absorbance decreased at 500, 550, and 700 nm. In the control ppt of potato starch, the half-life increased with the decrease in the wavelength, supporting the idea that the liberated amylose was hydrolyzed successively, decreasing the glucose chain length. The half-life at each wavelength was longer in the ppt-2 than in the control ppt, suggesting that proC1 itself could inhibit the hydrolysis of liberated amylose. In addition, the degree of the increase in the half-life by proC1 increased with a decrease in the wavelength. This suggests that the proC1-dependent inhibition might become more efficient in the shorter amylose produced from the liberated amylose than the liberated amylose itself. Such an inhibition was supposed to be possible (i) if amylose molecules liberated from the ppt-2 were combined with proC1, and (ii) if the shorter amylose molecules formed by hydrolysis of the liberated amylose were still combined with proC1 (Figure 1C). Such a strong combination might be formed by the binding of proC1 to the inside of the helical structure of amylose. 

In the ppt-1 of potato starch, the half-life at each wavelength was also longer than that of the control ppt, but the increase in the half-life was smaller in the ppt-1 than in the ppt-2 at any wavelength (Table 1). This suggests that the degradation products of proC1, which might be produced during the preparation of the ppt-1, were not so effective as proC1 for the inhibition. 

There is a possibility, however, that the slow starch hydrolysis was due to the proC1-dependent inhibition of α-amylase activity in pancreatin. To exclude this possibility under the conditions of this study, starch hydrolysis was studied by decreasing the concentration of pancreatin in the reaction mixture using the control ppt prepared from potato starch. The half-lives at 500, 550, and 700 nm increased with the decrease in pancreatin concentration from 10 μg/mL to 7 μg/mL, and the degrees of the increase at the three wavelengths were similar to each other; for example, the half-lives in the presence of 8 μg of pancreatin per mL were 35 ± 9, 31 ± 11, and 38 ± 18% (*n* = 3) longer at 500, 550, and 700 nm, respectively, than the half-lives in the presence of 10 μg of pancreatin per mL. The above result suggests that the slowdown of potato starch hydrolysis by proC1 might not be due to the inhibition of α-amylase activity by the procyanidin under the conditions of this study.

The half-lives of corn starch ppt-1 and ppt-2 at 500 and 550 nm but not at 700 nm were longer than that of the control ppt, and the increases were small (Table 1). The small increase indicates that the inhibitory effects of proC1 were not as significant in corn starch as in potato starch.

### 2.3. Effects of ProC1 on the Ratio of A_500_/A_700_

**Figure 2:**
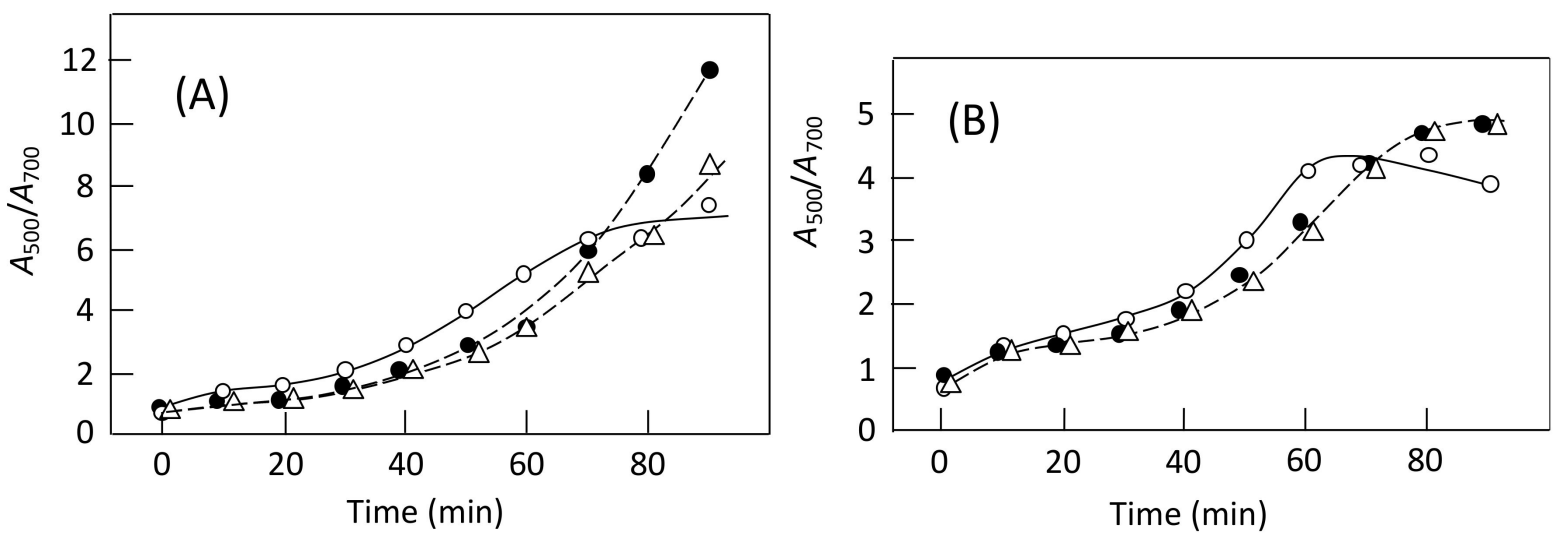
Changes in ratio of *A*_550_/*A*_700_ of starch-iodine complexes as a function of incubation time. The ratio was calculated using the data for Table 1. (**A**) Potato starch; (**B**) corn starch. (○) Heated without proC1; (●) proC1 added before heating; (△) proC1 added after heating. Each data point is the average of three experiments.

Figure 2A shows the ratio of absorbance at 700 nm (*A*_700_), compared to that at 500 nm (*A*_500_), as a function of the incubation time in potato starch. The absorbances at 700 and 500 nm roughly reflected the concentration of longer amylose molecules and shorter amylose molecules, respectively. The increase in ratio (*A*_500_/*A*_700_) during the initial 30 min of the incubation was somewhat faster in the control ppt than in the ppt-1 and the ppt-2. The increase in the ratio, however, was significantly faster in the control ppt (○) than the ppt-1 (●) and the ppt-2 (△) after 30 min of incubation. This result coincides with the result in Table 1 that the hydrolysis of amylose liberated from the control ppt was faster than that of amylose liberated from the ppt-1 and the ppt-2. The increase in ratio of the control ppt slowed down gradually after 60 min of incubation, suggesting that a considerable part of the liberated amylose had been hydrolyzed during the incubation period of 60 min. On the other hand, the ratio (*A*_500_/*A*_700_) increased exponentially after the incubation period for 30 min in the ppt-1 (●) and the ppt-2 (△). The exponential increase suggests that the shorter amylose formed from both ppts was not as easily further hydrolyzed as the shorter amylose molecules formed from the control ppt, supporting the idea presented in Figure 1C that shorter amylose/proC1 complexes were less hydrolyzable. 

The changes in the ratio (*A*_500_/*A*_700_) as a function of incubation time were also studied using the control ppt in the presence of 7–10 μg of pancreatin per mL; no significant effects of pancreatin concentration were observed for the time-course of the changes in ratio, supporting the idea that the difference in the increase in the ratio in the ppt-1 and ppt-2 from the control ppt was not due to the inhibition of α-amylase activity by proC1 under the conditions of this study.

In corn starch, there was no significant difference in the increase in the ratio (*A*_500_/*A*_700_) during the initial 40 min of incubation between the control ppt, ppt-1, and ppt-2 (Figure 2B). After 40 min of incubation, the increase in the ratio became faster in the control ppt (○) than in the ppt-1 (●) and the ppt-2 (△), indicating that proC1 could inhibit the hydrolysis of starch liberated from corn starch. The increase in the ratio of the control ppt slowed down after 60 min of incubation, and then the ratio seemed to decrease, while the ratio increased in the ppt-1 (●) and the ppt-2 (△) (Figure 2B). The decrease in the control ppt suggests that almost all starch molecules liberated from the ppt might have been hydrolyzed to shorter starch molecules during the 60 min of incubation, and then the shorter starch molecules formed were further hydrolyzed to shorter starch molecules that had small absorbance at 500 nm when complexed with iodine. The slow increase in the ratio in corn starch ppt-1 and ppt-2 after 60 min of incubation coincided with smaller effects of proC1 on the half-life of the hydrolysis of starch liberated from ppt-1 and ppt-2 (Table 1). If the hydrolysis of shorter amylose molecules was inhibited by proC1, as shown in Figure 1C, the smaller effects of proC1 on corn starch than potato starch suggest that the molar ratio of proC1 to leached amylose might be greater in corn starch than in potato starch (see Section 3). It has been reported that the amylose in corn starch can more effectively leach out during heating than in potato starch [16].

### 2.4. Binding of ProC1 Added to Starch before and after Heating

Table 2 (left side) shows the amounts of cyanidin formed from ppt-1 and ppt-2 prepared using potato starch and corn starch, and from the supernatants accompanying the preparation of the above ppts. The amount of cyanidin formed from the ppt-2 was about twice that formed from the ppt-1 in potato starch. The greater amount was correlated with the slower starch digestion in the ppt-2 than in the ppt-1 (Table 1). On the other hand, there was no significant difference in the amounts between the ppt-1 and the ppt-2 prepared from corn starch (Table 2). This result coincides with the data in Table 1 in that there were no significant differences in the slowdown of reducing sugar formation and the elongation of half-life between the ppt-1 and the ppt-2. The above results indicate that the slowdown of starch hydrolysis by proC1 might be related to the amount of proC1 bound to leached amylose.

The amount of cyanidin formed from the supernatants of ppt-2s was approximately 190 nmol, and the amount formed from the supernatants of ppt-1s was approximately 50% of the amount of the supernatants of ppt-2s (Table 2), indicating that proC1 was degraded during the heating with potato starch and corn starch. The degradation products included reddish components with an absorption peak around 500 nm in the reaction mixture (0.1 M of sodium phosphate (pH 7.0) with 0.15 M of NaCl). Some of the reddish components were bound to potato starch and corn starch. The amounts of cyanidin formed in supernatants of ppt-2 of potato starch and corn starch (approximately 190 nmol) suggest that the yield of cyanidin from proC1 was approximately 0.95. The initial amount of proC1 in the mixture to prepare the supernatant of ppt-2 was 200 nmol.

### 2.5. Amounts of ProC1 Bound to Starch Liberated from Potato Starch

We investigated whether starch liberated from the ppt-1 and the ppt-2 was combined with proC1 or not, using potato starch. After the incubation of the ppt-1 or the ppt-2 with and without pancreatin, precipitates (PPTs) and supernatants (SUPs) were prepared (Scheme 1B). Cyanidin was formed from the PPTs, and the amounts were decreased by pancreatin; the decrease became clear when compared in each pair of experiments (numbers in parentheses) (Table 3). The decrease might be explained by the pancreatin-induced liberation of starch molecules, combined with proC1 from the ppt-1 and the ppt-2.

The SUPs were supposed to contain starch molecules according to Figure 1. Then, the SUPs were mixed with methanol (final concentration: 80%) to obtain the precipitates (PPT-1s) and the supernatant (SUP-1s) (Scheme 1B). Cyanidin was formed from the PPT-1s and the formation was increased by pancreatin independent of the origin of PPT-1s (Table 3). This result indicates (i) that starch/proC1 complexes that were not soluble in 80% methanol could be diffused out from the ppt-1 and ppt-2 in the absence of pancreatin, and (ii) that pancreatin could liberate the starch/proC1 complexes. 

The amounts of cyanidin formed from SUP-1s were much greater than the amounts formed from PPT-1s, and the amounts in the SUP-1s prepared from the ppt-2 were greater than those in the SUP-1s prepared from the ppt-1 (Table 3). Taking the result in Table 1—namely, significantly slower hydrolysis of amylose liberated from the ppt-2 than the ppt-1—into account, the results in Table 3 suggest that, in addition to proC1 being strongly bound to amylose as forms not to be extracted with 80% methanol, proC1 extractable with 80% methanol also contributed to slow down amylose hydrolysis. The extractable proC1 might be able to reversibly combine with amylose to inhibit hydrolysis. It has been reported that a procyanidin dimer, oligomers, and polymers can bind noncovalently to the starch, inhibiting amylose hydrolysis [6,7,8,9], and that some other polyphenols can assemble two amylose molecules [23,24,25].

### 2.6. Effects of ProC1 on Hydrolysis of Reagent Amylose

If proC1 could inhibit the hydrolysis of amylose liberated from potato starch and corn starch (see above), proC1 might also be able to inhibit the hydrolysis of reagent amylose. Table 4 shows the hydrolysis of ppt-1, ppt-2, and control ppt prepared using proC1 and two types of amylose reagents (Mw4500 and Mw31200). Pancreatin induced the formation of reducing sugar in the ppt-1 and the ppt-2 prepared using Mw4500 (28 glucose residues in average), and the formations in the former and the latter were slower and faster, respectively, than that in the control ppt. The half-lives of the hydrolysis of the ppt-1 were approximately 50%, 40%, and 25% longer than those of the control ppt at 500, 550, and 700 nm, respectively, whereas the half-lives of the ppt-2 were similar to and somewhat shorter than those of the control ppt at the above wavelengths. These results indicate that components formed from proC1 by heating but not proC1 itself could slow down the hydrolysis of Mw4500. As a reason for the ineffectiveness of proC1, almost all of the Mw4500 in the ppt-2 was not combined with proC1. This idea was supported by the molar ratio of proC1 to Mw4500 in the ppt-2; the ratio calculated using the data from Table 2 (right side) was approximately 1:180. 

By the heating of proC1 with Mw4500, approximately 80% of proC1 was degraded (Table 2, right side). The degradation accompanied the formation of reddish components with an absorption peak around 500 nm, and some of the reddish components were combined with Mw4500 (data not shown). This might suggest the contribution of the degradation products of proC1 to inhibit the hydrolysis of the ppt-1 of Mw4500. The degree of the increase in half-life of the ppt-1 relative to the control ppt became greater with the decrease in wavelength from 700 to 500 nm (Table 4). The greater increase at shorter wavelengths might also be explained by Figure 1C. It has been reported (i) that reddish components, which are formed during the heating of procyanidin B2 with a reagent amylose (average molecular weight of 2600) and nonglutinous rice flour, are combined with amylose, and (ii) that their combining results in the slow hydrolysis of amylose [10,11]. 

Pancreatin-induced reducing sugar formation of the control ppt of Mw31200 (192 glucose residues in average) was approximately 70% slower than that of the control ppt of Mw4500, and the half-life of the former ppt was approximately twice as long as that of the latter ppt at 700 nm (Table 4), suggesting that Mw31200 was less hydrolyzable than Mw4500. The slower hydrolysis might be due to the presence of Mw31200, not dissolved during the heating, and of the greater double-helical structures in the control ppt of Mw31200 than in the control ppt of Mw4500. It has been reported that α-amylase is less accessible to amylose double helices [20,26].

The reducing sugar formation in ppt-1 and ppt-2 of Mw31200 was approximately 20% and 25% slower, respectively, than that of the control ppt. The half-lives of the ppt-1 of Mw31200 were 23% and 10% longer on average than those of the control ppt at 550 and 700 nm, respectively, whereas those of the ppt-2 were 46% and 20% longer on average than those of the control ppt at 550 and 700 nm, respectively. The slower hydrolysis of ppt-2 than ppt-1 (approximately half as fast) prepared using Mw31200 and the greater amount of proC1 bound to the ppt-2 than the ppt-1 (approximately twice as much) (Table 2) suggest that proC1 itself was more efficient at inhibiting the hydrolysis of Mw31200. The greater increase in the half-life at 550 nm than at 700 nm by proC1 suggests that the hydrolysis of shorter amylose produced from Mw31200 was less hydrolyzable than Mw31200 itself, as discussed for the effects of proC1 on the amylose hydrolysis that was liberated from potato starch (Figure 1C).

**Figure 3:**
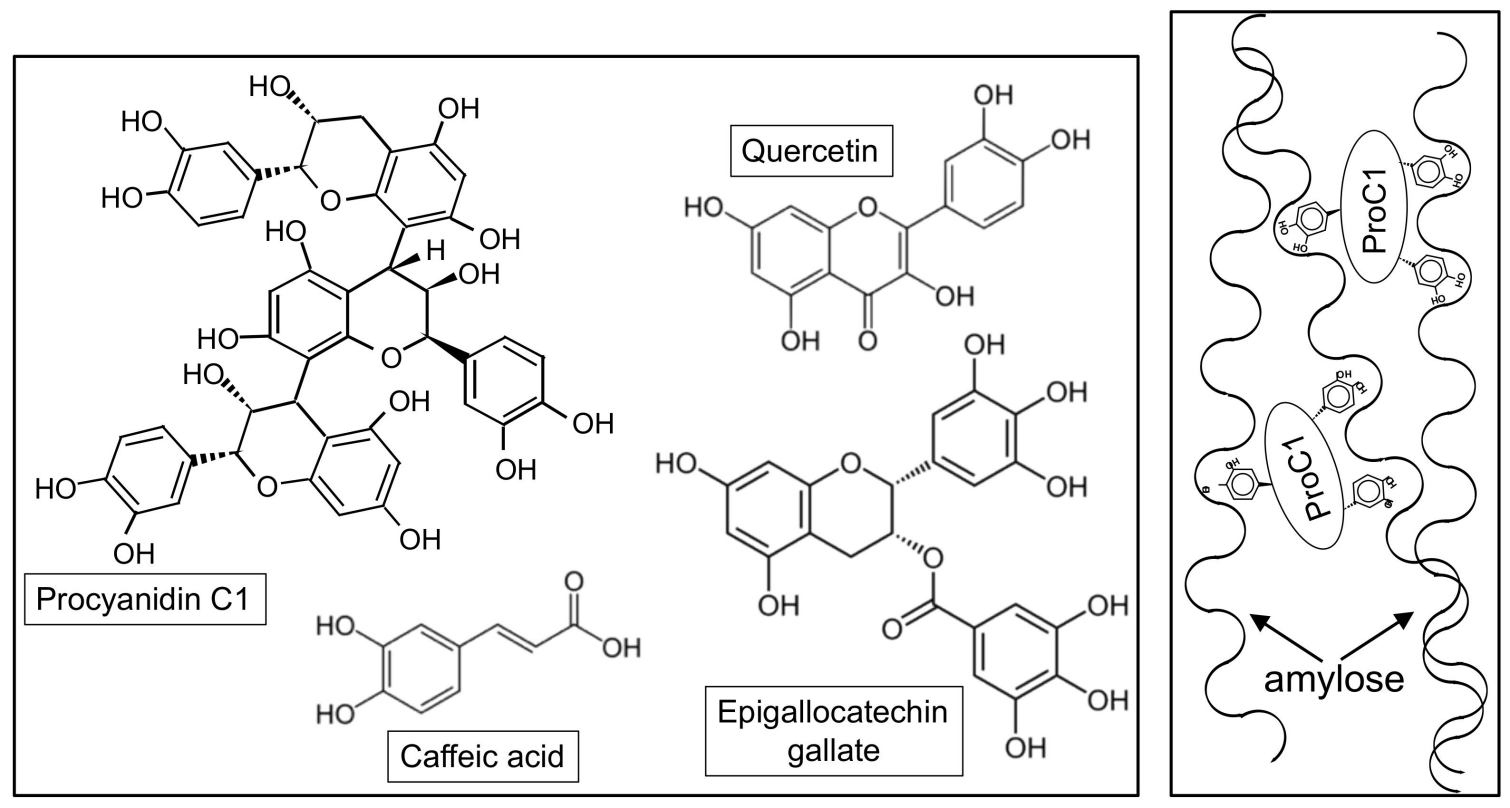
(**Left**) Chemical structure of phenolic compounds to be able to combine two amylose chains. The two-dimensional structure of proC1 was produced with reference to the structure in PubChem, and the other two-dimensional structures are from Wikipedia. (**Right**) Postulated amylose/proC1 complexes.

Then, the molar ratios of proC1 and Mw31200 in the ppt-2 were calculated using the data from Table 2. The value was approximately 1:20; approximately 5% of Mw31200 molecules were combined with proC1. The degree of the slowdown by proC1 (Table 3) was, however, greater than the slowdown expected from the molar ratio of proC1 to Mw31200. As a mechanism of the increase in inhibition, the proC1-dependent combining of two amylose molecules, increasing double-helical structures, is possible, according to reports that caffeic acid, quercetin, epigallocatechin gallate, etc. can assemble two amylose molecules [27,28,29,30]. Figure 3 shows a possible interaction of proC1 with Mw31200; three B-rings of flavan-3-ol of proC1 residues might combine two amylose molecules by binding to the helical structure of the amylose molecules. The combining of two Mw31200 molecules might increase double-helical structures, resulting in a greater slowdown of the hydrolysis expected from the molar ratio of proC1 to Mw31200.

## 3. Discussion

The different effects of proC1 between the two types of amylose reagents could be attributed to the difference in the molar ratio of proC1 to amylose molecules (see above), suggesting that the different effects of proC1 between potato starch and corn starch might also be attributed to the difference in the molar ratio of proC1 to leached amylose molecules. 

The molar ratios could be estimated using the data from Table 2 under the assumptions that (i) the starch contents in potato starch and corn starch flours are 81% and 86%, respectively [31], (ii) the amylose content in the starch is 25% [32], and (iii) proC1 may bind to amylose and amylopectin equally. Taking the molecular weight of native amylose of potato starch (7.9–10.2 × 10^5^, average 8 × 10^5^) and corn starch (1.5–2.9 × 10^5^, average 2.2 × 10^5^) [33] into account, the molar ratios of proC1 to amylose could be calculated as 1:0.8 and 1:0.4 for potato starch ppt-1 and ppt-2, respectively, and 1:2.6 and 1:2.5 for corn starch ppt-1 and ppt-2, respectively, on average. The molecular weight of amylose leached from potato starch and corn starch was supposed to be 2–5-fold smaller than native amylose according to [8,18,33]. Then, the molecular weights of leached amylose were estimated to be 1.6–5.1 × 10^5^ (average: 3.3 × 10^5^) and 0.3–1.5 × 10^5^ (average: 0.9 × 10^5^) for potato starch and corn starch, respectively. If almost all of the amylose was leached out from potato starch and corn starch during the preparation of ppts, the molar ratios of proC1 and amylose could be calculated as 1:1.9 and 1:1.0 for potato starch ppt-1 and ppt-2, respectively, and 1:6.3 and 1:6.1 for corn starch ppt-1 and ppt-2, respectively, on average.

Therefore, the different effects of proC1 on the inhibition of starch hydrolysis between potato starch and corn starch (Table 1) could be postulated to be due to the difference in the molar ratio. If one molecule of proC1 can assemble two amylose molecules, as in Figure 3, almost all of the amylose that leached out from the ppts of potato starch might be combined with proC1, while approximately 30% of leached amylose from ppt-1 and ppt-2 of corn starch might be combined with proC1.

If the inhibition of amylose hydrolysis by proC1 was dependent on the molecular weight of amylose, as discussed above, it could be postulated that the proC1-dependent inhibition of the hydrolysis of amylose liberated from nonglutinous rice flour was not so significant as potato starch was, because the molecular weight of rice amylose (1.5–1.8 × 10^5^) was similar to that of corn amylose [33]. In fact, proC1 could not efficiently slow down amylose hydrolysis when added to heated rice flour under the conditions in Table 1 (data not shown). From the present study, it became clear that the effects of procC1 on amylose hydrolysis were dependent on the molar ratio of proC1 to amylose molecules. To further understand the function of procyanidins in the intestine, the relationships between the polymerization degree of procyanidins and the molecular weight of amylose should be studied from the point of view of amylose hydrolysis. 

## 4. Materials and Methods

### 4.1. Reagents and Ingredients

Potato starch (product name: katakuri-ko) and corn starch sold as food were obtained from a local market. The potato starch was made by ÆON Co., Ltd. (Chiba, Japan), and the corn starch was made by Hinokuni Food Co., Ltd. (Kumamoto, Japan). Two types of amylose reagents, the average molecular weights of which were 4500 (Mw4500) and 31200 (Mw31200), were obtained from Glico Nutrition Co., Ltd. (Osaka, Japan). Procyanidin C1 (proC1) and cyanidin were from Funakoshi (Tokyo, Japan). 4-Hydroxybenzhydrazide and porcine pancreatin were from FUJIFILM Wako Pure Chemicals (Osaka, Japan). According to the producer’s data, the activities of digestive enzymes in the pancreatin are as follows: protease (26−46 units mg^−1^, pH 8), α-amylase (3−5 units mg^−1^, pH 7), and lipase (0.75−1.4 units mg^−1^, pH 8). Iodine solution (100 mM) was prepared as reported previously [12].

### 4.2. Preparation of Starch/ProC1 Complexes

Three test tubes, which contained potato starch or corn starch (10 mg each), were suspended in 1 mL of 0.1 M of sodium phosphate (pH 7.0), with 0.15 M of NaCl and 0.4 mM of proC1 added to one of the tubes. These tubes were heated in gently boiling water for 30 min. The test tube with proC1 turned reddish. The heated test tubes were left at room temperature for 10 min, and then 0.4 mM of proC1 was added to one of the two test tubes heated without porC1. After leaving the three test tubes for another 10 min, 5 mL of methanol (final methanol concentration, 80%) was added. The starch suspensions in 80% methanol were left for 10 min and then centrifuged at 1830× *g* for 5 min to prepare the supernatants and the precipitates. Hereafter, the precipitates prepared by heating the starch without and with proC1 are referred to as “control ppt” and “ppt-1,” respectively, and the precipitate prepared by adding proC1 to the heated starch is referred to as “ppt-2” (Scheme 1A) [14]. The prepared precipitates were suspended in 1 mL of 0.1 M of sodium phosphate (pH 7.0) with 0.15 M of NaCl to measure the pancreatin-induced hydrolysis of starch. Before measuring the hydrolysis, the absorption spectra of the methanolic supernatants and the suspensions of control ppt, ppt-1, and ppt-2 were measured to characterize the components in the supernatants and the precipitates.

Hydrolysis of starch in the suspensions of control ppt, ppt-1, and ppt-2 was initiated by adding pancreatin (10 μg/mL) at 37 °C. During the incubation, aliquots (10 and 25 μL) of the reaction mixture were withdrawn at 10 min intervals for 50 and 90 min, respectively. The former aliquot was used to estimate the reducing sugar formation and the latter to estimate the starch hydrolysis. In addition, the control ppt of potato starch, which was suspended in the above buffer solution with 0.15 M of NaCl, was incubated with 7, 8, 9, or 10 μg of pancreatin per mL to understand the effects of the decrease in α-amylase activity on the kinetics of starch hydrolysis.

Two types of amylose reagents (Mw4500 and Mw32100) (10 mg) were also treated as in Scheme 1A using 0.2 mM of proC1. Control ppt, ppt-1, and ppt-2 prepared from each reagent amylose were suspended in 1 mL of 0.1 M of sodium phosphate (pH 7.0) with 0.1 M of NaCl. Amylose hydrolysis was initiated by adding 5 μg of pancreatin per mL. Aliquots (10 and 25 μL) of the reaction mixture were withdrawn every 10 min to estimate the reducing sugar formation and amylose hydrolysis.

### 4.3. Measurement of Reducing Sugar Formation and Starch Hydrolysis

Reducing sugar formation was estimated by the method reported by Lever [34]. An aliquot (10 μL) of each reaction mixture, which was withdrawn as described above, and 0.15 mL of 0.33 M of 4-hydroxybenzhydrazide dissolved in 0.6 M of HCl were added successively to 1.35 mL of solution I (the mixture of 0.042 M of sodium citrate, 0.007 M of calcium chloride, and 0.5 M of sodium hydroxide), and then the mixture was heated in boiling water for 6 min. The absorbance spectrum of the supernatant was measured after centrifugation for 5 min at 1830× *g*. The rate of the reducing sugar formation of each sample was estimated from the absorbance increase at 410 nm as a function of incubation time, and the slope was determined by the least squares method. The effects of proC1 were expressed relative to their controls.

To estimate the starch hydrolysis, an aliquot (25 μL) of each reaction mixture, which was withdrawn as described above, and 0.1 mL of 100 mM of iodine solution were added successively to 1 mL of 0.1 M of sodium phosphate (pH 7.0) with 0.15 M of NaCl. After leaving it for 10 min at room temperature (about 25 °C), the absorption spectrum of the supernatant was measured. The starch hydrolysis was a first-order-like reaction. Then, the effect of proC1 could be estimated from the half-life.

### 4.4. Amount of ProC1 Bound to ppt-1 and ppt-2

Potato starch or corn starch (5 mg each) suspended in 0.5 mL of 0.1 M of sodium phosphate (pH 7.0) with 0.15 M of NaCl was heated with and without 0.4 mM of proC1 in boiling water for 30 min, and 0.4 mM of proC1 was added to the starch heated without proC1 as described in Scheme 1A (Section 4.2). Methanol (2 mL) was added to the three test tubes and centrifuged at 1830× *g* for 5 min after the incubation for 10 min at room temperature. The amounts of proC1 in the precipitates (control ppt, ppt-1, and ppt-2) and the respective supernatants were estimated from the cyanidin formation from proC1 as follows [35]: 2 mL of 1-butanol, 0.065 mL of 2% ammonium iron(III) sulfate dissolved in 2 M of HCl, and 0.2 mL of concentrated HCl were added in this order to an aliquot of the supernatants (1 mL) and the precipitates suspended in 0.5 mL of the above buffer solution with 0.15 M of NaCl, and then the mixtures were heated in gently boiling water for 30 min. The heated mixtures were washed with 1 mL of H_2_O to remove the yellowish ammonium iron(III), and then the concentrations of cyanidin in the 1-butanol fraction were estimated from the absorbance at 545 nm using standard cyanidin. 

The binding of 0.2 mM of proC1 to 5 mg of reagents amylose (Mw4500 and Mw31200) was also studied. Their control ppt, ppt-1, and ppt-2 were prepared and the amounts of proC1 bound and not bound to the ppt-1 and ppt-2 were estimated as described above.

### 4.5. Amount of ProC1 Bound to Starch Liberated from ppt-1 and ppt-2 of Potato Starch

The ppt-1 or ppt-2 prepared using 5 mg of potato starch and 0.2 mM of proC1 were suspended in 0.5 mL of 0.1 M of sodium phosphate (pH 7.0) with 0.15 M of NaCl. The suspensions were incubated with and without pancreatin (20 μg/mL) for 20 min at 37 °C, and then centrifuged at 7700× *g* for 5 min (Scheme 1B). The precipitates (PPTs) were suspended in 0.5 mL of the above buffer solution with 0.15 M of NaCl. The supernatants (SUPs) were transferred to test tubes to precipitate the starch by adding 2 mL of methanol. After leaving to stand for 5 min, the methanolic mixture was centrifuged at 1830× *g* for 5 min to prepare the supernatants (SUP-1s) and the precipitates (PPT-1s). The PPT-1s and the SUP-1s would contain proC1 not extracted and extracted with 80% methanol, respectively. The PPTs and PPT-1s suspended in the above buffer with 0.15 M of NaCl (0.5 mL) and an aliquot (1 mL) of the SUP-1s were used to estimate the amounts of proC1, as described in Section 4.4.

### 4.6. Spectrophotometric Measurements

All spectrophotometric measurements were performed using UV-2450 combined with a sphere integrating apparatus (ISR-240A) (Shimadzu Corporation, Kyoto, Japan). The path length of the measuring beam was 2 mm if not otherwise specified.

### 4.7. Statistical Analysis

Each experiment was repeated 3–4 times. The data were presented as averages with standard deviations. Significant differences between the samples were determined using the Student’s *t*-test with the significance threshold set to 0.05.

## Figures and Tables

**Table 1:** Effects of proC1 on pancreatin-induced hydrolysis of liberated starch.

Origin of Starch	Precipitates	Half-Life (min) ^1^		
		500 nm	550 nm	700 nm
Potato (*n* = 3)	control ppt	40.0 ± 1.0 (100)	25.2 ± 2.7 (100)	16.1 ± 3.3 (100)
	ppt-1	48.0 ± 4.2 (119 ± 10) *	27.5 ± 1.4 (109 ± 6) *	16.4 ± 3.1 (101± 4)
	ppt-2	56.5 ± 0.8 (141 ± 2) *	32.5 ± 1.2 (130 ± 19) *	18.8 ± 1.8 (119 ± 15) *
Corn (*n* = 3)	control ppt	41.5 ± 1.2 (100)	27.6 ± 2.7 (100)	19.3 ± 0.9 (100)
	ppt-1	46.4 ± 4.0 (111 ± 7) *	31.3 ± 2.0 (113 ± 6) *	19.2 ± 0.6 (100 ± 8)
	ppt-2	45.5 ± 2.1 (109 ± 2) *	30.3 ± 1.5 (110 ± 6) *	19.1 ± 0.3 (99 ± 5)

^1^ Half-lives were determined as described in Section 4.3; values in parentheses: half-life relative to the control ppt in each paired experiment. * *p* < 0.05 relative to half-life of the control ppt in parentheses at each wavelength.

**Table 2:** The amounts of proC1 bound to potato starch, corn starch, and amylose reagents.

Precipitates ^1^	Cyanidin Formed (nmol per 5 mg of Starch) (*n* = 3)			
	Potato Starch ^2^	Corn Starch ^2^	Mw4500 ^3^	Mw31200 ^3^
control ppt	0	0	0	0
ppt-1	6.3 ± 0.1	7.5 ± 0.3	4.0 ± 0.3	4.1 ± 0.2
ppt-2	11.8 ± 0.7	7.9 ± 0.4	6.2 ± 1.2	7.9 ± 0.4
sup of control ppt	0	0	0	0
sup of ppt-1	101 ± 14	109 ± 11	18.9 ± 1.4	17.9 ± 1.6
sup of ppt-2	192 ± 18	186 ± 15	93.8 ± 6.1	76.2 ± 6.4

^1^ Please see Scheme 1A for the preparation of ppts. “sup of each ppt” was the supernatant obtained when each ppt was prepared by the centrifugation. ^2^ The initial amount of proC1 was 200 nmol. ^3^ The initial amount of proC1 was 100 nmol.

**Table 3:** Binding of proC1 to precipitates prepared from ppt-1 and ppt-2 of potato starch.

	Pancreatin	Cyanidin Formed (nmol per 5 mg of Starch) (*n* = 3)		
		PPT ^2^	PPT-1 ^2^	SUP-1 ^2^
ppt-1 ^1^	without with	1.68 ± 0.07 (100%) 1.31 ± 0.29 * (77 ± 14%) *	0.75 ± 0.06 1.24 ± 0.30 *	3.27 ± 0.25 4.04 ± 0.71
ppt-2 ^1^	without with	2.22 ± 0.30 (100%) 1.79 ± 0.32 (80 ± 7%) *	0.72 ± 0.12 1.02 ± 0.13 *	10.1 ± 0.6 11.6 ± 0.8 *

^1^ These ppts were prepared using potato starch. ^2^ Prepared by incubation of ppt-1 and ppt-2 with and without pancreatin. Values in parentheses: Cyanidin formed “with pancreatin” relative to “without pancreatin” in each pair of experiments. * *p* < 0.05 relative to “without pancreatin.”

**Table 4:** Effects of proC1 on the hydrolysis of reagent amylose.

Amylose	Precipitate	Formation of Reducing Sugars (%) ^1^	Half-Life (min)		
			500 nm	550 nm	700 nm
Mw4500 (*n* = 4)	control ppt	100	53.8 ± 1.2 (100)	39.2 ± 3.8 (100)	24.7 ± 1.4 (100)
	ppt-1	81 ± 9	81.3 ± 8.7 (151 ± 19) **	53.7 ± 2.9 * (138 ± 8) **	30.7 ± 1.4 * (124 ± 4)
	ppt-2	122 ± 9	55.5 ± 0.8 (103 ± 3)	39.1 ± 2.3 (100 ± 5)	22.2 ± 1.2 * (91 ± 4)
Mw31200 (*n* = 3)	control ppt	31 ± 2	– ^2^	69.4 ± 1.6 (100)	58.3 ± 0.9 (100)
	ppt-1	25 ± 5	– ^2^	86.0 ± 4.1 * (123 ± 8) **	68.6 ± 9.8 * (110 ± 4)
	ppt-2	23 ± 2	– ^2^	101.9 ± 16 * (146 ± 16) **	82.7 ± 8.8 * (120 ± 6)

^1^ Relative to control ppt of Mw4500. ^2^ Difficult to estimate because of great deviations. Values in parentheses: half-life relative to the control ppt in each paired experiment. * *p* < 0.05 relative to “control ppt”; ** *p* < 0.05 relative to half-life at 700 nm.

## Data Availability

The data presented in this study are available on request from the corresponding author.

## Sample Availability

Samples of procyanidin C1 are not available from the authors.

## Abbreviations

Control ppt	precipitate prepared by heating starch without proC1
proC1	procyanidin C1
ppt-1	precipitate prepared by heating starch with proC1
ppt-2	precipitate prepared by adding proC1 to heated starch
